# A Clinical Prospective Observational Cohort Study on the Prevalence and Primary Diagnostic Accuracy of Occult Vertebral Fractures in Aged Women with Acute Lower Back Pain Using Magnetic Resonance Imaging

**DOI:** 10.1155/2017/9265259

**Published:** 2017-05-25

**Authors:** Atsushi Terakado, Sumihisa Orita, Kazuhide Inage, Go Kubota, Tomohiro Kanzaki, Hiroshi Mori, Yuji Shinohara, Junichi Nakamura, Yusuke Matsuura, Yasuchika Aoki, Takeo Furuya, Masao Koda, Seiji Ohtori

**Affiliations:** ^1^Kitachiba Spine and Sports Clinic, Chiba, Japan; ^2^Department of Orthopaedic Surgery, Graduate School of Medicine, Chiba University, Chiba, Japan

## Abstract

**Background:**

Elderly female patients complaints of acute low back pain (LBP) may involve vertebral fracture (VF), among which occult VF (OVF: early-stage VF without any morphological change) is often missed to be detected by primary X-ray examination. The current study aimed to investigate the prevalence of VF and OVF and the diagnostic accuracy of the initial X-ray in detecting OVF.

**Method:**

Subjects were elderly women (>70 years old) complaining of acute LBP with an accurate onset date. Subjects underwent lumbar X-ray, magnetic resonance imaging (MRI), and bone mineral density (BMD) measurement at their first visit. The distribution of radiological findings from X-ray and magnetic resonance imaging (MRI) as well as the calculation of the prevalence of VF and OVF are investigated.

**Results:**

The prevalence of VF among elderly women with LBP was 76.5% and L1 was the most commonly injured level. Among VF cases, the prevalence of OVF was 33.3%. Furthermore, osteoporotic patients tend to show increased prevalence of VF (87.5%). The predictive values in detecting VF on the initial plain X-ray were as follows: sensitivity, 51.3%; specificity, 75.0%; and accuracy rate, 56.7%.

**Conclusions:**

Acute LBP patients may suffer vertebral injury with almost no morphologic change in X-ray, which can be detected using MRI.

## 1. Introduction

Elderly female patients complaining of acute low back pain (LBP) may suffer from vertebral fractures (VF), sometimes especially occult vertebral fractures (OVF) that are not detected in the primary radiological examination with no apparent morphological change. OVF can result in late-onset neurological deterioration if not diagnosed quickly and correctly. However, its prevalence is still unclear, with only retrospective data provided by previous studies. These retrospective studies reported a prevalence of 6.5–14.1% in the elderly population [1–3]. These data are based on radiographic modalities with various diagnostic rates, with 87% for plain radiography and 98% for magnetic resonance imaging (MRI) [2]. However, no studies have provided prospective data on the prevalence of OVF.

In addition, osteoporosis-related VF is known to cause functional disorders in 30–50% of women over the course of their lifetime [4] and greatly increases the burden of OVF. However, its prevalence is also unclear.

The current study aimed to prospectively investigate the prevalence of VF in elderly women complaining of LBP with exact onset by examining the prevalence of OVF detected using MRI, as well as the diagnostic accuracy of plain radiography in detecting OVF. Also the prevalence of OVF in osteoporotic patients was investigated.

## 2. Methods

Subjects were patients with acute LBP visiting our clinic between November 2011 and March 2012. All patients were informed of the risks and study methodology following the Declaration of Helsinki, as reported in a previous related study [1]. The inclusion criteria included the following: (1) female aged 70 years or older; (2) being diagnosed with acute LBP with an accurate onset to within 2 weeks; and (3) being available for MRI scanning. Exclusion criteria included the following: (1) poor quality of the initial radiograph; (2) being not available for MRI scanning; and (3) the presence of pathologies that tend to show a vertebral intensity alteration in MRI such as tumor, infection, and hematologic disease.

All the patients underwent radiological examination on their first visit to detect any evidence of VF and its associated properties. The radiological examination included a lumbar plain X-ray in sitting position and MRI (1.5-Tesla system; Toshiba, Japan). Protocols involved a T1-weighted and a short tau inversion-recovery (STIR) pulse sequence in the sagittal plane to evaluate intensity changes in the vertebrae. The present study regarded intensity changes in the MRI as the absolute incidence of vertebral fractures based on evidence of a diagnostic accuracy of almost 100% for vertebral fractures [2, 5], including a bone bruise without deformity [6] as well as apparent morphological fracture. Bone mineral density (BMD) measurement using dual-energy X-ray absorptiometry (DXA) was also performed at the first visit to diagnose osteoporosis using the criteria of osteoporosis with a* T*-score ≤−2.5 SD. Three experienced senior orthopedic doctors prospectively examined and analyzed the radiographical features in a blind manner with no clinical information. Specifically, morphological changes indicating VF on the plain X-ray and intensity changes in the MRI were analyzed.

## 3. Results

**Figure 1:**
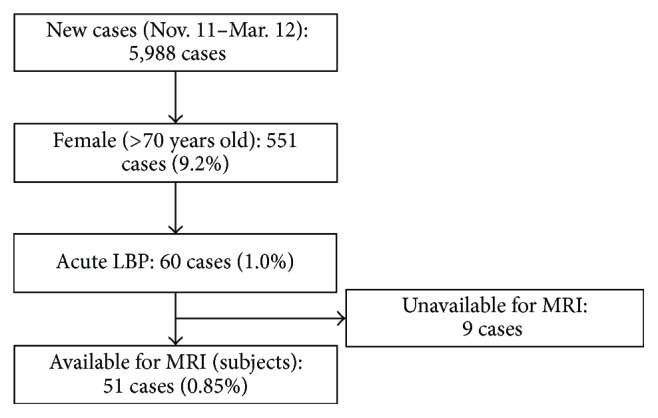
Patient selection flowchart. Among the total of 5,988 new outpatients, 551 (9.2%) were female (>70 years old). Acute LBP patients with exact origins amounted to 60 cases (1.0%). Among these, 9 cases were unavailable for MRI due to medical contraindication. The final 51 cases were included in the current study.

**Figure 2:**
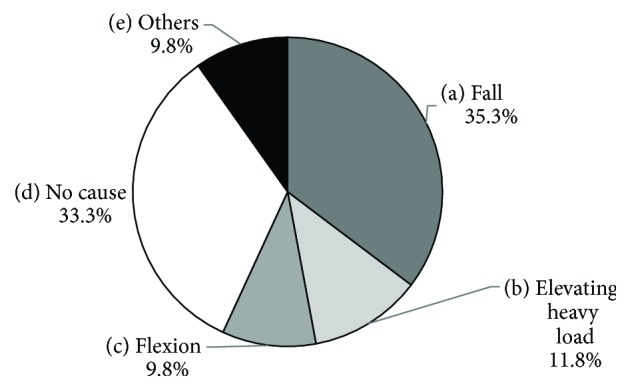
Onset of LBP. Apparent injury cases amounted to 47.1% (a + b), while no cause and light loading cases amounted to 52.9% (c + d + e).

**Figure 3:**
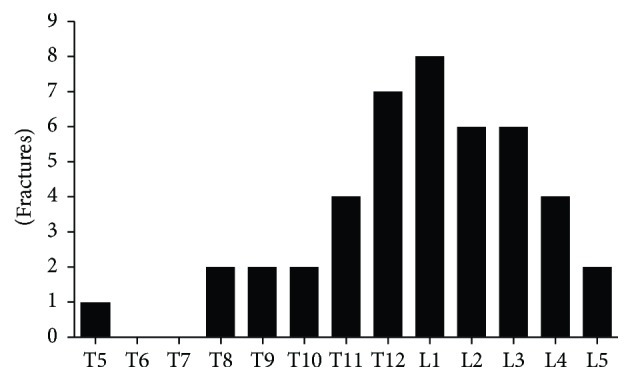
Fracture levels detected using MRI. The most common level was the L1 vertebra, followed by T12, L2, and L3.

Figure 1 depicts the patient selection flowchart. The total number of outpatients was 69,463, with 5,988 new cases during the study period. Elderly female patients (≥70 years old) amounted to 551 cases, among whom those with acute LBP amounted to 60. Finally, 51 subjects were included in the study, as 9 patients with LBP were unavailable for MRI.  Figure 2 depicts the distribution of the onset of LBP. Subjects with a history of apparent injury or heavy loading amounted to 47.1% (24/51), while no cause or light loading cases amounted to 52.9% (27/51). The most common VF level was L1, followed by T12, L2, and L3 (Figure 3).

**Figure 4:**
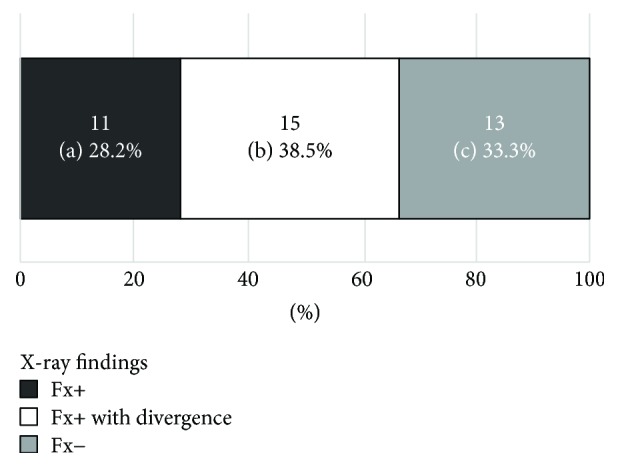
Coincidence between the X-ray findings and MRI findings. Among the cases with fractures confirmed using MRI, 28.2% ((a) 11/39) showed a positive fracture finding both on X-ray and on MRI, while 38.5% ((b) 15/39) showed divergence between these two radiological modalities. The remaining 13 cases ((c) 33.3%) showed a positive MRI finding with a negative X-ray finding, suggesting a true occult fracture (no deformity or fracture in the plain X-ray with positive MRI findings).

**Figure 5:**
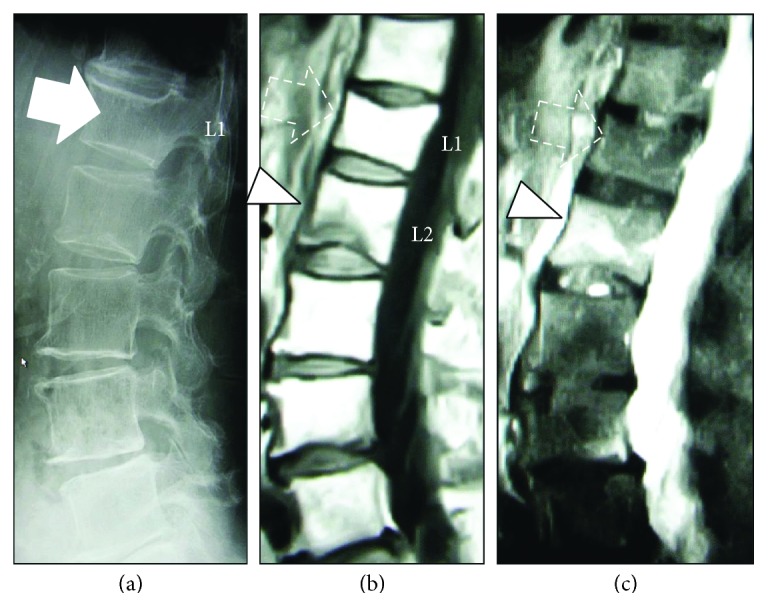
A 71-year-old woman with osteoporosis (*T*-score <−2.5 SD) and a history of no falls/injuries. (a) Plain lumbar radiography showed a possible L1 fracture (solid arrow). (b) T1-weighted MRI showed a compression fracture at the caudal adjacent L2 vertebral body (arrowheads) with a high intensity change in the T2-STIR image (c) not at the presumed level (dotted arrow) (c).

**Figure 6:**
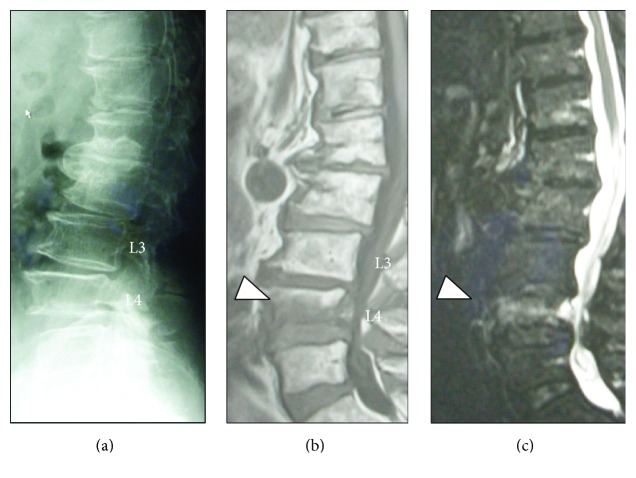
A 79-year-old woman with preosteoporosis (*T*-score = −2.0 SD) and a history of strain. Plain lumbar X-ray showed spondylosis with no apparent fracture (a). MRI showed a low intensity lesion in the T1-weighted image (b) with a high intensity change in the T2-STIR image (c) indicating an occult fracture at the L4 vertebral body (arrowheads).

The distribution of the radiological findings is summarized in Table 1. The mean *κ*-value for the interobserver error was calculated as 0.77. The prevalence of VF as indicated by intensity changes in the MRI scan was 76.5% (39/51). Among these VF cases, 28.2% (11/39) showed a positive fracture finding on both X-ray and MRI, while 38.5% (15/39) showed divergence between these two modalities. The remaining 13 cases (33.3%) showed a positive MRI finding with a negative X-ray finding, suggesting the presence of OVF, with no deformity or fracture in the plain X-ray with positive MRI findings (Figure 4). Among the 51 subjects, 24 cases were diagnosed with osteoporosis. Twenty-one of those with osteoporosis (87.5%) had VF and 6 (28.6%) had OVF. Two representative cases are presented in Figures 5 and 6.

Based on Table 1, the predictive values of detection for VF using plain X-ray were calculated as follows: sensitivity, 51.3% (20/39); specificity, 75.0% (9/12); false negatives, 48.7% (19/39); false positives, 25% (3/12). Regarding diagnostic accuracy, the positive predictive value was 86.7% (20/23), the negative predictive value was 32.1% (9/28), and accuracy was 56.7%  [(20 + 9)/51].

## 4. Discussion

The present study prospectively investigated the prevalence of VF in patients with acute LBP, especially with a focus on OVF without radiologic collapse in elderly women (≥70 years old). We prospectively demonstrated that the prevalence of VF was 76.5% with the most common injured level being L1. Among the patients with VF, the prevalence of OVF was 33.3%. About 53% of the patients experienced VF with no history of injuries. Furthermore, osteoporotic patients with acute LBP showed an increased prevalence of VF of 87.5%. In addition, the present study demonstrated that the initial plain radiography examination had moderate predictive values in detecting VF.

OVF is clinically important not only because it is a musculoskeletal injury but also because it can lead to late-onset risks such as pseudarthrosis and neurological disorders. A pseudarthrosis derived from OVF can often develop minor and major abnormalities at the level of the overlooked lesion, which can cause neurological deterioration due to a late diagnosis [7]. In cervical cases, the risk of neurologic sequelae has been reported to be 10 times higher in patients with an occult injury than in those who have an injury identified during the initial screening [8, 9]. Lumbar lesions can also cause late-onset neurologic disorders after overlooked OVF.

Previous studies have reported the prevalence of OVF to be 6.5–14.1% [1–3, 6], which is much lower compared to that of the present study (33.3%). One potential reason for this difference can be the study design; the previous studies were retrospective, whereas the present one is prospective. Retrospective studies for OVF prevalence may easily overlook some of the cases; in contrast, the present study demonstrated a higher rate of OVF by picking up each case. Regardless of the underlying cause, the diagnosis of VF largely relies on a morphologic collapse in, for example, the anterior bone cortex [6]. However we have to keep in mind that the initial plain radiography was shown to have relatively low sensitivity (51.3%), negative predictive value (32.1%), and accuracy (56.7%). Retrospective studies using plain radiography alone have shown underdiagnostic accuracy of 66% in OVF [10] which is higher than that in the present prospective study. Therefore, the true accuracy may be lower than that previously reported. That indicates that in case the patients complain of persistent back pain, especially movement-related (flexion, extension, and rotation) and lasting pain, physicians should consider a functional X-ray with flexion/extension considering OVF. Furthermore, the L1 vertebra is statistically the most feasible location for fracture [11], which is consistent with the results from the current study. These facts may help primary physicians to diagnose OVF when using a plain X-ray with a relatively low rate of accuracy.

When OVF is suspected, other radiological modalities such as computed tomography (CT) and MRI should be considered. MRI is the best recognized modality for its ability to detect bone bruises after trauma, which is considered to represent a combination of trabecular microfracture, edema, and hemorrhage [12, 13]. The usefulness of MRI in diagnosing spinal fractures has been discussed in previous reports. Wang et al. reported the usefulness of MRI in detecting spinal trauma as follows: occult fractures in the anterior column (sensitivity 100%); cord deformity (100%); tearing of the posterior longitudinal ligament (71%); ligament tears in the posterior column and soft tissue disruption (100%); fracture in the thoracic spinous process (100%); and facet fracture (100%) [5]. Thus, we regarded the sensitivity of MRI in detecting VF as 100% in the current study. Furthermore, meta-analyses have concluded that MRI is an effective tool when combined with CT scanning in detecting OVF [14, 15], while CT scan alone can miss OVF [14]. MRI is also useful in showing avascular necrosis and occult fractures better than conventional radiographs or CT scans [5].

The present study showed an increased prevalence of VF (87.5%) and OVF (28.6%) in osteoporotic patients with acute LBP. These patients are feasible candidates for VF; however, elderly women without osteoporosis can experience VF even when no morphological fractures are diagnosed using plain radiography, as seen in the present study. New radiological techniques may help in this situation, such as an anisotropic study and the subsequent assessment of color and vector maps providing a noninvasive tool for assessing the risk of fracture due to osteoporosis [16].

The present study has some limitations. First, the sample size was small, although there were 6,000 primary outpatients. Future studies should include a mass cohort study. Second, the study may have included patients with a preexisting altered intensity in their vertebrae before their initial visit; however, the chances are minimal. To our knowledge, there are no data suggesting a prospective radiological examination with routine X-ray and MRI for acute LBP patients at their first visits. Third, the final diagnosis of fracture was mainly made by the intensity changes using MRI, and the detailed pain profiles such as the relationship between the pain level and the existence of fractures were not obtained. This is one of the issues that should be clarified in the future study.

In conclusion, the present prospective study demonstrates that primary physicians must be aware that elderly patients with acute LBP may involve a vertebral injury with a prevalence rate of 76.5%, among which occult fractures amount to 33.3%. Most patients suffered VF with no injuries. Initial plain radiography has a relatively moderate predictive value in detecting VF; thus, MRI can be helpful. Furthermore, acute LBP patients with osteoporosis tend to show an increased prevalence of VF.

## Figures and Tables

**Table 1:** Radiological distributions.

		MRI		
		IC+	IC−	
X-ray	Fx+	20	3	23
	Fx−	19	9	28

		39	12	51

Fx: fracture with morphological change. IC: intensity change.
